# Neonatal Bone Disorders

**DOI:** 10.3389/fped.2021.602552

**Published:** 2021-04-06

**Authors:** Vrinda Saraff, Ruchi Nadar, Nick Shaw

**Affiliations:** ^1^Institute of Applied Health Research, University of Birmingham, Birmingham, United Kingdom; ^2^Department of Endocrinology & Diabetes, Birmingham Women's and Children's National Health Service (NHS) Foundation Trust, Birmingham, United Kingdom; ^3^Institute of Metabolism and Systems Research, University of Birmingham, Birmingham, United Kingdom

**Keywords:** newborn, bone, mineralization, structural, antenatal

## Abstract

Neonatologists care for newborns with either an antenatal suspicion or postnatal diagnosis of bone disease. With improved ultrasound imaging techniques, more cases of neonatal bone disorders are identified antenatally and this requires further diagnostic/molecular testing either antenatally or soon after birth for confirmation of the diagnosis and facilitating subsequent management. Prompt diagnosis is vital in certain conditions where initiation of treatment is time critical and life saving. We outline an approach to diagnosis, investigation, and management of a neonate with a suspected bone disorder.

## Introduction

The fetus accrues 80% of bone mineral content between 24 weeks gestation and term (1). Neonatal bone disorders encompass a spectrum of bone conditions resulting from either structural or mineralization defects, a process that has already commenced in the fetus (2). Good quality fetal ultrasound images are likely to detect bone abnormalities in the second trimester onwards (3). This is followed by molecular confirmation of the diagnosis in some cases and postnatally in the rest. Early diagnosis and effective management can be vital to the impact this can have on childhood, adolescent, and adult bone heath. In this manuscript, we have outlined an approach to diagnosis, investigation, and management of neonatal bone disorders.

## Approach to Neonatal Bone Disorders

Fetal ultrasonography has been used since the 1950's to estimate gestational age, detect multiple pregnancies and diagnose fetal anomalies (4). Occasionally antenatal ultrasound evaluation raises suspicion of a bone disorder in the fetus (5). For example, severe osteogenesis imperfecta can be diagnosed antenatally based on multiple long bone fractures and limb deformities. Also fetal femora or humeri length of less than the 5th centile or −2 SD from the mean in a second trimester scan often raises the suspicion of skeletal dysplasia (6). However more often antenatal fetal radiographs show a constellation of features which may be common to several disorders. Equally, in many cases, antenatal imaging may have been completely normal. Based on postnatal x-ray findings and biochemistry, bone disorders can be largely categorized into either a predominantly structural bone defect (with normal bone biochemistry) or a mineralization bone defect (with abnormal bone biochemistry). This classification aids further targeted investigations.

## Structural Bone Defects

### Osteogenesis Imperfecta

Osteogenesis Imperfecta (OI) is a genetic disorder of increased bone fragility and low bone mass which has a wide spectrum of severity. It has an incidence of 1 in 10–20,000 births and occurs with equal frequency in genders and ethnic groups.

The majority are due to defects in the amount or quality of Type 1 collagen which is coded for by two genes, COL1A1 and COL1A2. These are autosomal dominant in inheritance accounting for ~90% of cases of OI. Autosomal recessive forms have been recognized in the past 20 years and account for between 5 and 10% of cases. These are due to a variety of different genes that predominantly affect the synthesis of Type 1 collagen.

The traditional classification of OI described four types with Type I the mildest and most frequent form, Type III a severe form with multiple fractures and bowing deformity of limbs and Type IV an intermediate form of moderate severity (7). These three types are associated with long term survival. An additional form, Type II was described, also known as perinatal lethal as affected babies would die in the neonatal period due to respiratory insufficiency. As a consequence of the discovery of new forms of OI in recent years, revised classifications have been proposed (8, 9). However most specialists who manage affected individuals like to categorize them as mild, moderate or severely affected.

#### Clinical Presentation

There are several ways in which a baby with OI may present in the neonatal period.

##### Severe Forms

Antenatal detection at the time of routine ultrasound scans is increasingly identifying severe forms *in utero*. Skeletal abnormalities such as bowed limbs, fractures, and small chest size may lead to a precise diagnosis of OI or a broader description as a lethal skeletal dysplasia which is only recognized as OI after birth.

**Figure 1:**
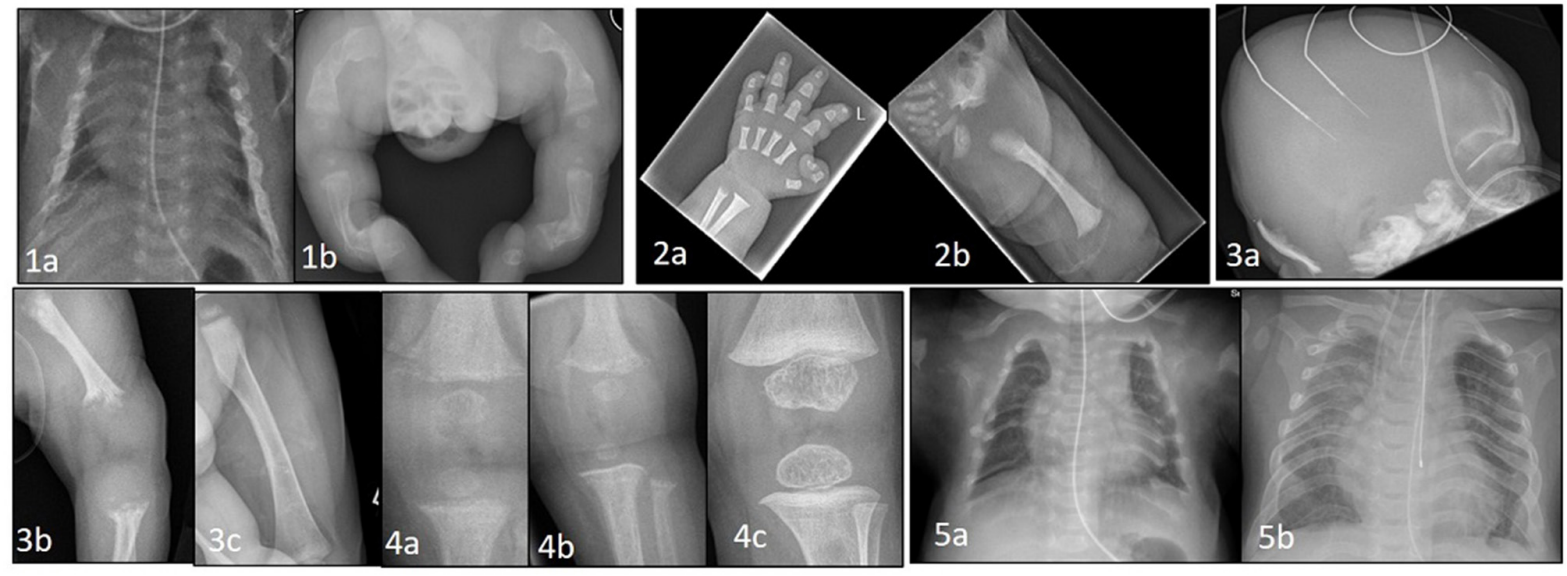
**(1a)** Thin, gracile and beaded ribs in a case of severe osteogenesis imperfecta at birth. **(1b)** Lower limbs showing bowing and deformity of long bones with multiple fractures in various stages of healing. **(2a)** Hand x-ray in achondroplasia showing short metacarpals and phalanges with trident sign. **(2b)** Hip shows classic features of horizontal acetabular roof, squared off iliac crest, small sacrosciatic notch, and scalloping of proximal femur. **(3a)** Perinatal hypophosphatasia at birth showing severe skull hypomineralization. **(3b)** severe hypomineralization of long bones and metaphyseal lucencies. **(3c)** Healing of metaphyseal lesions and markedly improved mineralization after 12 months of asfotase alfa treatment. **(4a)** Knee x-ray in a case of NHSPT at 3 months showing metaphyseal irregularity, coarsening of trabeculae, and subperiosteal resorption. **(4b)** progressive changes at 6 months, and **(4c)** complete healing following total parathyroidectomy. **(5a)** Chest X-ray at birth in a baby with TRPV6 mutation. Ribs are malformed with severe demineralization. **(5b)** Spontaneous improvement at 2 years of age with remodeling, improved mineralization of ribs and thoracic volume.

If not identified in the antenatal period, some severely affected infants with small deformed chests due to the presence of multiple rib fractures (Figures 1-1a) will develop respiratory insufficiency within a few days of birth requiring assistance ranging from supplemental oxygen to positive pressure ventilation. More commonly, a severely affected baby will present at birth with evidence of short stature, bowed limbs and multiple fractures (Figures 1-1b). Subsequent investigation with reviews of skeletal surveys by experienced clinicians will usually lead to a diagnosis of OI which can be confirmed by genetic testing.

##### Mild and Moderate Forms

These may present in the neonatal period in several ways. There may be evidence of a short bowed femur which might have been detected on an antenatal scan. It is often not apparent that this is due to OI until the child starts to fracture, which may not be for some months or years after birth. An alternative presentation is with a dislocated or unstable hip due to the ligamentous laxity known to be characteristic of OI. An occasional, but uncommon, presentation is when an affected baby presents in the neonatal period with a long bone fracture, which leads to the identification of other fractures (including rib fractures) on skeletal survey.

#### Assessment

There are a number of investigations of importance when a baby presents with a suspected diagnosis of OI. It is important to perform a skeletal survey including the skull and spine with review by a pediatric radiologist looking for features such as Wormian bones (accessory skull bones completely surrounded by suture lines), rib and vertebral fractures and bowing deformity of long bones. Additional imaging of the cervical spine and brain may be indicated in severely affected infants for the presence of cervical spine abnormalities which may compromise spinal cord function, or hind brain abnormalities such as basilar invagination.

All infants with OI should have blood tests to check bone profile, renal function, and vitamin D levels to ensure these are normal if bisphosphonate treatment is being considered. Severely affected babies are likely to be cared for on a neonatal unit with routine assessment of respiratory function.

Genetic testing is now readily available in many countries with initial testing for abnormalities in COL1A1 and COL1A2 which will account for 90% of cases. If the results of these are normal consideration should then be given to testing for the recessive forms of OI (10). Although the results will not be available for several weeks they are important not only for confirmation of the diagnosis but for genetic counseling to the parents for future pregnancies.

#### Management

A severely affected baby will be managed initially on a neonatal intensive care unit. There is no reason why milk feeds cannot be commenced if there is no significant respiratory distress. This will often be *via* a nasogastric tube initially, as a continuous feed with progression to bolus feeds if well-tolerated. Breast feeding can also be attempted if there is no significant respiratory distress. Many severely affected infants will still require nasogastric feeding at the time of hospital discharge. An important aspect of nutrition in such babies is the recognition that they are short and therefore it is not appropriate to expect their weight gain to be within the normal centiles (11). Such a practice may lead to overfeeding and excess weight gain.

The input of a multidisciplinary OI specialist team is important in management and will often be undertaken by this team visiting the neonatal unit to review the baby and meet the parents. Such support is also important for the staff of the neonatal unit who may not be familiar with managing such babies and will be uncertain of activities such as handling. The specialist OI team will educate the parents and staff about the condition with information such as likely prognosis and outcome, how to handle, dress, feed, wash, and transport the baby. They will continue as an appropriate resource to provide support until the baby is discharged and is followed up in their own specialist OI clinic. Advice about where to find additional information on OI from appropriate websites such as the Brittle Bone Society (www.brittlebone.org) in the UK will also be provided.

The use of bisphosphonate drugs (given intravenously as infusions every few months) has become an important component in the management of severely affected babies with OI in the past 20 years (12). There is evidence that bisphosphonates reduce the frequency but do not eliminate the risk of fractures. Intravenous Pamidronate has been the drug that has been most used, often given every 8 weeks in the first 2 years of life. There have been reports of the development of respiratory insufficiency in babies following the first infusion and so it is advisable to administer this whilst still on the neonatal unit (13). Hypocalcaemia is an infrequent occurrence and is more likely to occur if the baby is vitamin D deficient. Treatment of vitamin D deficiency and provision of a maintenance daily dose of at least 400 IU is important in such infants. Bisphosphonate infusions are usually given *via* peripheral veins although in some infants with difficult venous access a central line is required.

Some babies who are severely affected develop respiratory difficulty due to a small chest size secondary to pulmonary hypoplasia and in addition they can also have multiple rib fractures. Such infants would historically be considered to have perinatal lethal forms of OI and would not have survived. However, with respiratory support such as home oxygen and CPAP many of these infants are surviving and with time are capable of being weaned from such support.

It is important for parents of a new baby with OI that they receive appropriate support and information with an expectation that they will survive long term, have normal intelligence and will be able to attend a mainstream school.

### Other Skeletal Dysplasias

Skeletal dysplasias, a complex group of heritable disorders of the bone and cartilage affect the fetal skeleton as it develops *in utero*. They often present as congenital bowing of the long bones, particularly the femurs, detected in the second trimester ultrasound evaluation. Short long bones when compared against normative data can determine whether there is primary rhizomelic or mesomelic shortening (6).

The common angulated femur or bent bone dysplasias in the neonatal period as described in the Skeletal dysplasia registry include Campomelic disorders (24.4%) including Campomelic and Kyphomelic dysplasias, Thanatophoric dysplasias (23.9%), OI (18.1%), short rib dysplasia (10.2%), hypophosphatasia (3.5%), Type 2 collagen disorders (3.1%), Stuve Weidman dysplasia, and Achondroplasia (1.3%) amongst others (14). Some of these dysplasias, evident on antenatal ultrasound scans are lethal in the neonatal period owing to the small chest circumference and associated pulmonary hypoplasia.

#### FGFR3 Chondrodysplasias

Mutations in the FGFR3 gene lead to a spectrum of conditions ranging from the lethal Thanatophoric dysplasia to the milder hypochondroplasia (15, 16). FGFR3 expressed in chondrocytes and mature osteoblasts regulates bone growth (17).

##### Achondroplasia

Achondroplasia is the most common form of short limb dwarfism due to a mutation in the FGFR3 gene, with a prevalence of 1 in 25,000 individuals. It is inherited in an autosomal dominant pattern with 80% arising from new spontaneous mutations (18).

##### Clinical Presentation

In most cases, short limbs, hands and fingers, frontal bossing, depressed nasal bridge with a large head on second trimester antenatal ultrasound scans raises suspicion of Achondroplasia. The diagnosis is subsequently confirmed by molecular testing for FGFR3 mutation. Postnatally, the diagnosis is apparent at birth due to the rhizomelic shortening of limbs, frontal bossing, midfacial hypoplasia, and macrocephaly (19).

##### Management

**Figure 2:**
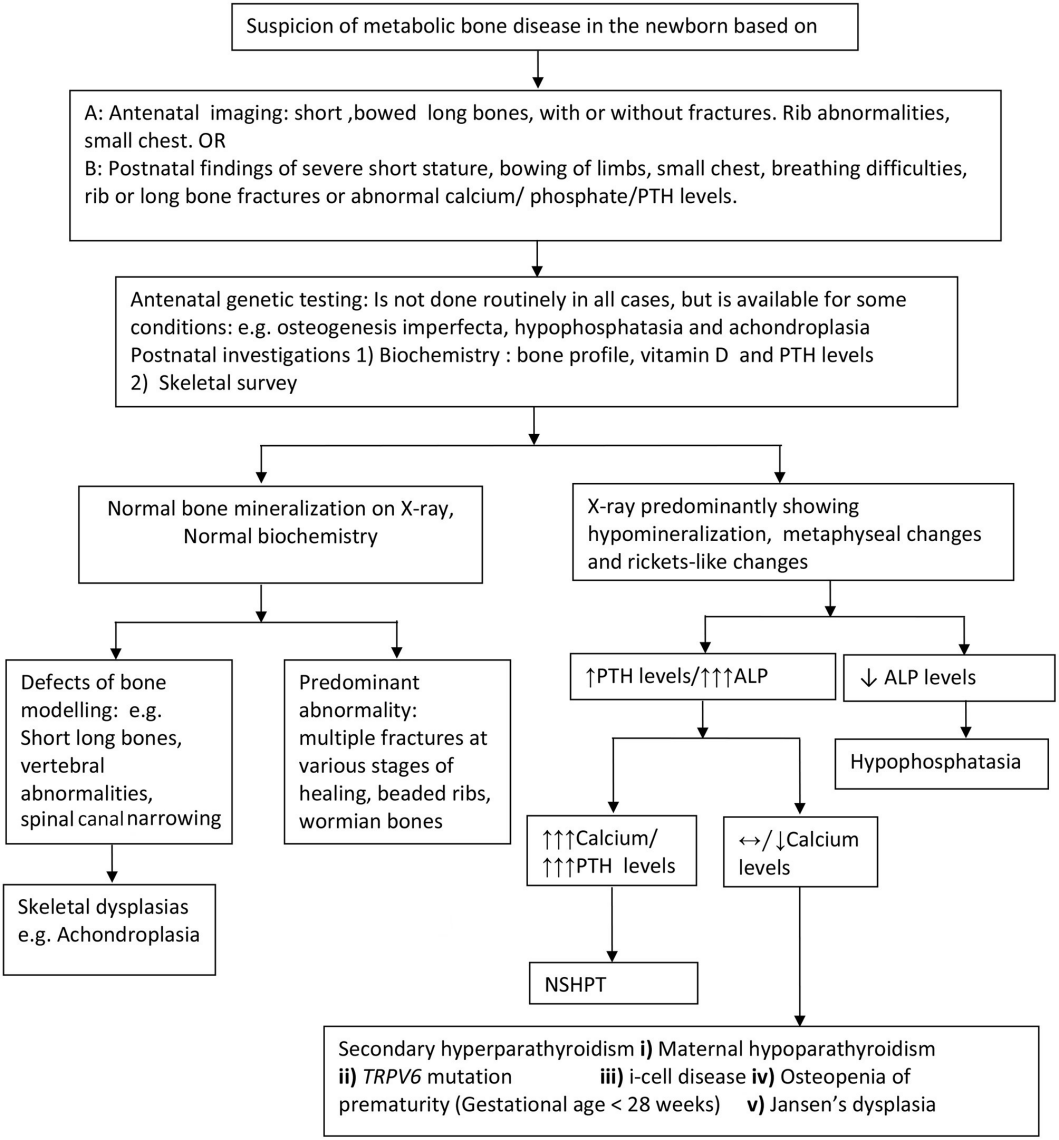
Approach to the diagnosis of metabolic bone disorders in the newborn. PTH, parathyroid hormone levels; ALP, Alkaline phosphatase; NSHPT, neonatal severe hyperparathyroidism; TRPV6, transient receptor potential cation channel, subfamily V, member 6.

If a diagnosis of Achondroplasia is suspected at birth, this can be confirmed by performing a skeletal survey (Figures 1-2a,b). Large calvaria, narrow foramen magnum, progressive reduction in the interpedicular distance in the lower spine, small trident pelvis are hallmark radiological features for Achondroplasia. MRI brain and spine is recommended to look for cervicomedullary compression occurring secondary to foramen magnum stenosis. Genetic confirmation by testing for FGFR3 mutations is also available. Bone biochemistry is often normal in these neonates (20, 21).

Treatment in Achondroplasia is mainly supportive. Some children might require neurosurgery to relieve cervical cord compression and others orthopedic intervention to help with limb deformities. Close follow up beyond the neonatal period is important with clinical assessment, history taking, and neurological examination. Because of the risk of sleep disordered breathing a sleep study is recommended in the first 6 months. With a better understanding of the molecular processes involved in Achondroplasia, various drug trials looking at blocking FGFR3 ligands, FGFR3 and its downstream signaling including tyrosine kinase inhibitors and C-type natriuretic peptide (CNP) analogs such as vosoritide, amongst others, are underway (22). Most advanced of the possible therapeutic options and the only study currently recruiting neonates, involves the use of CNP analogs in the BMN111 (Vosoritide) study by Biomarin.

## Bone Mineralization Defects

### Hypophosphatasia

Hypophosphatasia (HPP) is a rare heterogenous inherited metabolic bone disorder caused by a loss of function mutation in the ALPL gene (23), resulting in the lack of tissue non-specific alkaline phosphatase (TNSALP) activity. The more severe forms are predominantly inherited in an autosomal recessive pattern with an incidence of 1 in 100,000 live births. Deficiency in TNSALP activity results in the accumulation of inorganic pyrophosphate (PPi) which disrupts hydroxyapatite crystal formation and inhibits skeletal mineralization (24). The clinical spectrum of HPP can be variable and the two forms relevant to the neonatal period include Prenatal Benign and Perinatal Lethal form.

#### Clinical Presentation

Benign Prenatal HPP, as the name suggests, is a mild form of the condition with asymmetrical skeletal changes first noticed on prenatal ultrasonography, usually in the second trimester of pregnancy, including limb bowing, with or without skeletal hypomineralization and normal chest and abdominal circumference. The ultrasound appearances usually improve in the third trimester and they can run a variable postnatal clinical course ranging from the more severe infantile HPP (symptoms and signs apparent between 1 and 6 months of age) to the mild Odonto HPP where only teeth are affected with no skeletal manifestations (25, 26).

Perinatal HPP, the most lethal form, presents antenatally on fetal ultrasonography as short long bones, under mineralized skeleton, small chest, and abdominal circumference. It is apparent at birth with short deformed limbs, severely hypomineralized skeleton, small chest with hypoplastic lungs and, in some cases, pyridoxine deficiency seizures. Perinatal HPP can mimic hypoxic ischemic encephalopathy (27) and delay diagnosis in the absence of antenatal suspicion. Low serum ALP level in the presence of typical radiological features of tongue-like lucencies in the metaphysis, rickets-like changes, and hypomineralized skeleton (Figures 1-3a–c) should clinch the diagnosis of HPP (28).

#### Management

If HPP is suspected, serum ALP activity, plasma PLP, urine phosphoethanolamine levels and genetics for ALPL gene mutation alongside skeletal survey must be performed to confirm the diagnosis. Age- and sex- specific ALP activity should be used to prevent delay in the diagnosis of HPP. Prompt referral to the tertiary pediatric bone service is vital for further assessments and initiation of enzyme replacement therapy, Asfotase Alfa (Strensiq), which is time critical and life saving (28). Strensiq is delivered by subcutaneous injections three times a week for life. Follow up by a multidisciplinary team is also extremely important.

### Neonatal Hyperparathyroidism

Normally parathyroid hormone (PTH) secretion from the parathyroid gland is regulated to maintain serum calcium levels within the normal range. Any drop in calcium level is sensed by the G protein coupled calcium sensing receptor (CaSR) situated on the chief cells, resulting in increased PTH secretion. The reverse occurs in hypercalcemia.

Neonatal severe primary hyperparathyroidism (NSHPT) is a result of almost complete loss of parathyroid calcium sensing due to homozygous CaSR mutations, resulting in very high serum calcium with unsuppressed PTH levels. This is a rare autosomal recessive disorder which is potentially lethal, often occurring when there is consanguinity.

#### Clinical Presentation and Management

Newborns may be asymptomatic at birth, but present within days to weeks. The presentation may be delayed up to 6 months when failure to thrive, poor feeding, and hypotonia (29) become apparent. They have severe hypercalcemia (commonly >4 mmol/L) (30) and X-rays show demineralized bones, subperiosteal resorption, rib fractures, and changes of rickets (Figures 1-4a–c). In the majority of cases hypercalcemia is severe and total parathyroidectomy is the only definitive therapy. Bisphosphonates such as pamidronate are used in the short term to control hypercalcemia until total parathyroidectomy can be performed.

### Mucolipidosis Type II (i-cell disease)

This is an autosomal recessive condition caused by mutations in the GNPTAB gene that code for N-acetylglucosamine phosphotransferase complex which catalyze the post-translational modification of lysosomal enzymes. Impaired calcium transfer during pregnancy due to affected placenta is one of the postulated mechanisms for the metabolic bone disease seen in these babies. Imbalance between osteocyte and osteoclast function has been shown in mouse models (31).

#### Clinical Presentation and Management

Antenatal short femurs and intrauterine growth retardation have been described with radiological abnormalities detectable by 18–20 weeks of pregnancy (32). Newborns may manifest respiratory distress soon after birth (33, 34), hyperparathyroidism and fractures. X-rays show widespread osteopenia, sub-periosteal bone resorption, metaphyseal changes, shortening and undermodelling of long bones consistent with hyperparathyroidism. Biochemistry is characterized by elevated PTH and alkaline phosphatase levels with low or normal calcium levels. Diagnosis is confirmed by enzyme analysis and genetic studies for *GNPTAP* gene. Beyond the age of 6 months, typical manifestations become apparent with coarse facies, skeletal disproportion, and organomegally.

Homozygous TRPV6 mutations present with clinical, radiological and biochemical features identical to i-cell disease (Figures 1-5a,b). TRPV6 (the transient receptor potential cation channel, subfamily V, member 6) plays an important role in materno-fetal calcium transfer. Short long bones, bowed femora, rib deformities and Intrauterine Growth Restriction (IUGR) are reported antenatally (35). After birth the bone disease rapidly heals with enteral calcium supply available for mineralization. Radiological signs completely resolve by 18 months to 2 years of age.

### Miscellaneous Bone Conditions Presenting in the Neonatal Period

Transient neonatal hyperparathyroidism and bone disease similar to above situations, has been described in maternal hypoparathyroidism (36). In some cases, previously unknown maternal disease may be diagnosed after the birth of an affected baby. Osteopetrosis can present in the neonatal period with hypocalcaemia and high PTH with dense, osteosclerotic bones on X-ray. Osteopenia of prematurity is another condition that may manifest with pathologic rib and long bone fractures. As most materno-fetal calcium transfer during pregnancy occurs during the last trimester, babies born <28 weeks gestational age are particularly predisposed to this condition. A comprehensive review of the diagnosis and management of this condition has been published recently (37).

## Investigations

With advancement in technology and expertise, 3D antenatal ultrasonography and fetal MRI as an adjunct when spinal abnormalities are suspected, are increasingly used in prenatal diagnosis of potential bone disorders (38). Good quality antenatal imaging influences further targeted molecular genetic testing, invasive prenatal diagnosis in at risk families, antenatal counseling, informing obstetricians of the best mode of delivery and perinatal management.

Postnatal x-rays, with or without a complete skeletal survey, are the most useful investigation in diagnosing both structural (with normal bone biochemistry) and bone mineralization defects (with abnormal bone biochemistry) in the neonatal period. Radiological features suggesting hyperparathyroidism viz undermineralization, periosteal cloaking, metaphyseal changes, and subperiosteal resorption (diaphyseal tunneling) along with an elevated PTH, suggest primary or secondary hyperparathyroidism (Figure 2).

First line biochemical investigations include bone profile (including serum calcium, phosphate, and alkaline phosphatase levels), parathyroid hormone and 25 hydroxy vitamin D levels. When calcium levels are only marginally elevated, consider further investigations including maternal calcium and PTH profile, baby's urine for glycosaminoglycans and enzyme assay (for i-cell disease). Based on clinical, radiological, and biochemical abnormalities, targeted molecular genetics such as Type 1 collagen mutation, ALPL, FGFR3 CaSR, TRPV6, and GNPTAB, to name a few should be considered to confirm the diagnosis. In a few cases, despite extensive genetic investigations, the cause for the bone disorder may remain unknown.

Children with complex neonatal bone disorders should ideally be managed in a tertiary pediatric unit by a multidisciplinary team comprising of a pediatric endocrinologist, geneticist, radiologist, orthopedic and neurosurgeon, dentist, physiotherapist and occupational therapist, clinical psychologist, specialist bone nurses, and social worker to provide the necessary family support.

## Conclusion

Neonatal bone health is of growing interest not only due to the impact initial management can have on bone health during childhood, adolescence, and early adulthood but also the need for early and accurate diagnosis and initiation of life saving treatment such as enzyme replacement therapy in conditions such as hypophosphatasia. Neonatal bone disorders are a rapidly developing area of research interest with interventional studies and drug trials targeting bone health as early as the antenatal period in conditions such as osteogenesis imperfecta or in the neonatal period for Achondroplasia. However, the mainstay of managing neonatal bone disorders remains follow up by a specialist multidisciplinary team to achieve best possible functional outcomes.

## Author Contributions

All authors listed have made a substantial, direct and intellectual contribution to the work, and approved it for publication.

## Conflict of Interest

The authors declare that the research was conducted in the absence of any commercial or financial relationships that could be construed as a potential conflict of interest. The reviewer HM declared a past co-authorship with one of the authors NS to the handling editor.
